# Network Silsesquioxane-Based Organogel/Silicone Composites for the Long-Lasting Delivery of Nitric Oxide

**DOI:** 10.3390/molecules31081343

**Published:** 2026-04-19

**Authors:** Kyle D. Hallowell, Fatima Naser Aldine, Hope N. Vonder Brink, Ashley K. Mockensturm, Hitesh Handa, Elizabeth J. Brisbois, Alexis D. Ostrowski, Joseph C. Furgal

**Affiliations:** 1 Department of Chemistry and Center for Photochemical Sciences, Bowling Green State University, Bowling Green, OH 43403, USA; kyledh@bgsu.edu (K.D.H.); fnasera@bgsu.edu (F.N.A.); mockena@bgsu.edu (A.K.M.); 2 School of Chemical, Materials, and Biomedical Engineering, University of Georgia, Athens, GA 30602, USA; hhanda@uga.edu (H.H.); ejbrisbois@uga.edu (E.J.B.); 3 Pharmaceutical and Biomedical Sciences Department, College of Pharmacy, University of Georgia, Athens, GA 30602, USA

**Keywords:** nitric oxide (NO) release, silsesquioxanes, -RSNO, nitrosothiol, organogel, biomedical materials

## Abstract

Nitric oxide (NO) is a gaseous biocompatible radical molecule with demonstrated biomedical and antimicrobial benefits. Developing adaptable, long-lasting delivery systems for NO has become an essential goal for both combating resistant bacterial growth and providing sustained medical benefits. Silsesquioxane (SQ)-based organogels were chosen and synthesized as robust, tunable NO-release platforms. These highly stable SQ gel frameworks, composed of silicon–oxygen backbones with variable R groups, exhibited high porosity and surface area and offered chemical versatility, enabling control over NO loading and release. 3-Mercaptopropyl groups were utilized as sulfur-based NO-releasing substituents (-RSNOs), with additional R groups capable of altering accessibility to RSNO sites through hydrophobicity and steric hindrance. The NO release profile, rate, and duration of the functionalized gels were also tailored by adjusting the number of RSNO sites in the elastomeric system, thereby enabling a customizable release profile. This combination of NO-releasing silsesquioxanes with silicone elastomers yields composite materials that are integratable into biomedical applications, offering NO release up to 40 days within modeled physiological conditions in PBS buffer.

## 1. Introduction

Nitric oxide (NO) is a naturally occurring free radical crucial for many physiological and pathological processes in mammals, including bacterial defense, platelet adhesion, thrombosis, vasodilation, and wound healing [1,2,3,4,5]. Considerable research over the past two decades has centered on developing effective NO-generating and releasing materials for clinical and antimicrobial use. Though synthetic compounds like S-nitroso-N-acetylpenicillamine (SNAP), S-nitrosoglutathione, N-diazeniumdiolate, and nitrosyl, metal complexes [6,7,8] have been designed for controlled stabilization and release of NO; their practical use is limited by short release durations, poor shelf life, and rapid molecular breakdown [9,10,11].

Silica nanoparticles are being explored as biocompatible, efficient drug delivery systems [12,13] but may cause cytotoxicity or aggregates if not properly filtered from the body. To address these drawbacks, our research developed highly porous silsesquioxane (SQ)-based organogels that provide a stable, durable platform for sustained NO release and incorporation into advanced materials. We leveraged SQ’s flexible chemistry to introduce functional R groups that mimic natural sulfur–nitroso (-SNO) moieties, which are essential for NO donation. Prior studies show that silicone- and silica-based platforms can deliver NO effectively due to their high stability, biocompatibility, and customizable porosity, but many degrade quickly or deplete NO reservoirs, resulting in only short burst releases [14]. For instance, SNAP, when in solution, typically rapidly releases NO and loses efficacy over hours. Under physiologically relevant conditions (PBS buffer, pH~7.4, and 37 °C) and in the absence of metal chelators like EDTA, SNAP undergoes thermal decomposition with a half-life of approximately 6 h, corresponding to a roughly 50% decay of the donor over that interval [14,15]. Other systems suffer similar challenges: swift NO depletion, poor long-term performance, or limited customizability [16]. For this reason, many studies have focused on incorporating these materials into polymer matrices, particularly silicone rubber, to achieve sustained, long-term release while overcoming the challenges described above [17]. In most earlier reports, NO release was monitored in real time for only short periods, typically minutes rather than days. Specifically, NO flux and cumulative release were often followed for only around an hour after hydration at 37 °C. However, achieving true long-term, multiday release has remained challenging, especially when the NO donor is covalently incorporated into the material, as this often limits the ability to sustain extended-release profiles. Although this covalent attachment between the NO-releasing molecule and the polymer’s backbone enhances stability and prevents leaching, it often restricts the extended release of NO, making long-term, controlled delivery difficult to achieve [18].

**Figure 1:**
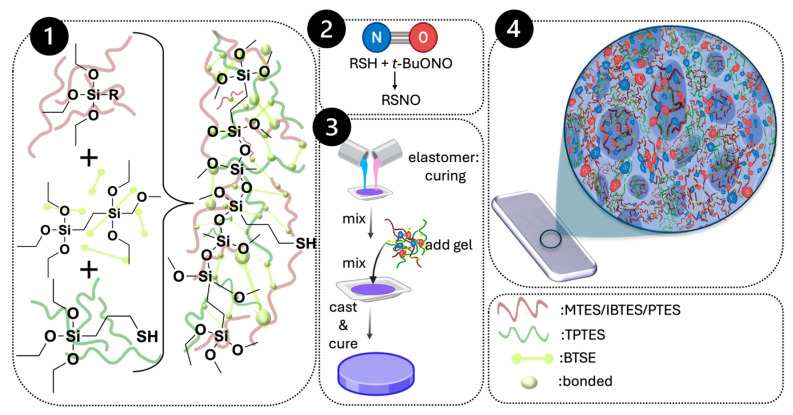
Polymer synthesis and elastomer curing process to create NO-releasing systems. 1. Reagents and Silsesquioxane Network Gel, 2. Loading of NO, 3. NO release composite formation, 4. NO release composite cast on a surface.

Our silsesquioxane organogel (SQ) framework (Figure 1) addresses these limitations, offering sustained, tunable NO release and easy integration into hybrid/polymeric systems. We shield SQ -RSNO groups with various hydrophobic and bulky substituents and encapsulate them in porous networks to test how the architecture affects NO release and fluid access. These approaches significantly extended the lifetimes of NO release, with optimized gels maintaining NO output for more than thirty days. The organogels, which are three-dimensional porous polymers [19,20,21], are suited to a range of medical devices due to their thermal and structural versatility. Structurally, SQ hybrid organogels consist of silicon–oxygen backbones linked by carbon bridges, with three distinct substituents that modulate their properties.

A key synthetic method adapts Hu et al.’s fluoride-catalyzed approach [22], replacing the R-triethoxysilane monomer with two types of R-triethoxysilane monomers in different ratios. Thiopropyltriethoxysilane (TPTES) imparts primary -RSNO functionality, while other monomers adjust hydrophobicity and bulkiness. Methyltriethoxysilane (MTES) ensures a robust methyl-protected base, and isobutyltriethoxysilane (IBTES) or phenyltriethoxysilane (PTES) further increases steric bulk/hydrophobicity to protect RSNO moieties and prolong NO release.

In forming these organogels, SQ molecules self-assemble into porous structures with pore characteristics governed by substituents, bridges, and synthesis conditions [22]. More surface area allows for higher NO loading, and the network protects RSNO groups—restraining direct exposure and prolonging release. Over time, the release stabilizes, enabling lasting NO delivery. Further adjustments to bridging and substituent species could unlock even more tunable architectures.

We then embedded the tailored SQ organogels in elastomeric polymer matrices, creating a stable platform for NO storage and controlled delivery [23]. Mimicking silicone-based systems, the composite maintained mechanical support and regulated NO diffusion from the gels. To avoid sulfur-catalyst poisoning [24], the silicone used tin-based dibutyltin dilaurate for curing at ambient conditions, preventing damage to the nitrosated organogels. Various loadings were evaluated to determine the optimal NO release duration and utility as an additive; this compatibility also suggests broader application in other elastomeric materials.

Meticulously adjusting organogel composition, elastomer loading, and surrounding R-group hydrophobicity, the resulting SQ system achieved continuous NO release for more than a month, outpacing previously reported silicone- or silica-based versions [25,26]. These results establish SQ-derived hybrid materials as robust, long-term vehicles for NO delivery in biomedical and protective settings. The goal of this study is to establish a series of silsesquioxane/siloxane-based NO-release materials and to investigate their structure–property relationships, laying the foundation for future biological effects and medical device applications.

## 2. Results and Discussion

Medical devices are becoming more efficient and affordable, improving patient outcomes. Silicone-based polymers are widely used in these applications due to their safety and versatility [23]. Our network organogels can act as -RSNOs and be fine-tuned by modifying their chemical structures to control stability and extend their NO-release duration. We have used pore size and hydrophobic shielding to adjust NO-release profiles, building on developments in the literature to help prevent infections and clotting over longer periods [27,28]. Our approach offers a powerful strategy to enhance the safety and performance of medical devices.

Fluoride rearrangement/catalysis is a well-documented procedure for creating sol–gel silicon-based polymers [29,30,31]. The synthesis of highly porous silsesquioxane-based organogels has been established by our group in solvents such as acetonitrile, in which full hydrolysis/condensation is achieved, and only R-group replacement was needed here to incorporate thiol functionalities [22,32]. A small amount of H_2_O was added to the solvent, as it was found to promote better substitution of Si-O-R molecules into Si-O-Si chains [33]. A series of organogel ratios, 3:1 to 6:1, were prepared with variable mmol proportions of methyltriethoxysilane (MTES) and thiopropyltriethoxysilane (TPTES). Short-duration NOA runs revealed that the 3:1 ratio organogel gave off a fluctuating, unsustained release rate compared to the 4:1 ratio. It was theorized that alterations in the gel matrix between 3:1 and 4:1 caused the unexpected release rate. The 5:1 and 6:1 ratio organogels were set aside due to their lower overall sulfur content, and the 4:1 ratio was ultimately chosen as the system of analysis for this study. For each, a 0.6 mol ratio of 1,2-bis(triethoxysilyl)ethane (BTSE) was used as a bridging molecule to improve porosity [22,32]. Once a precipitate was formed during the sol–gel process, the reaction was vacuum filtered and rinsed with solvent, or in the case of phenyltriethoxysilane (PTES) gels, concentrated first by rotary evaporation (40 °C and 220 mbar) due to fine particle formation before isolation. The resulting powders were dried overnight in a vacuum oven at 60 °C. The next day, the powders were crushed with a mortar and pestle and returned to the vacuum oven to evaporate any remaining liquids from the organogel. The naming for these organogels is as follows: methyl is NMS, iso-butyl is NIS, and phenyl is NPS (Table 1). The organogels were characterized as outlined below.

### 2.1. FTIR Spectral Analysis Confirming Chemical Modifications

**Figure 2:**
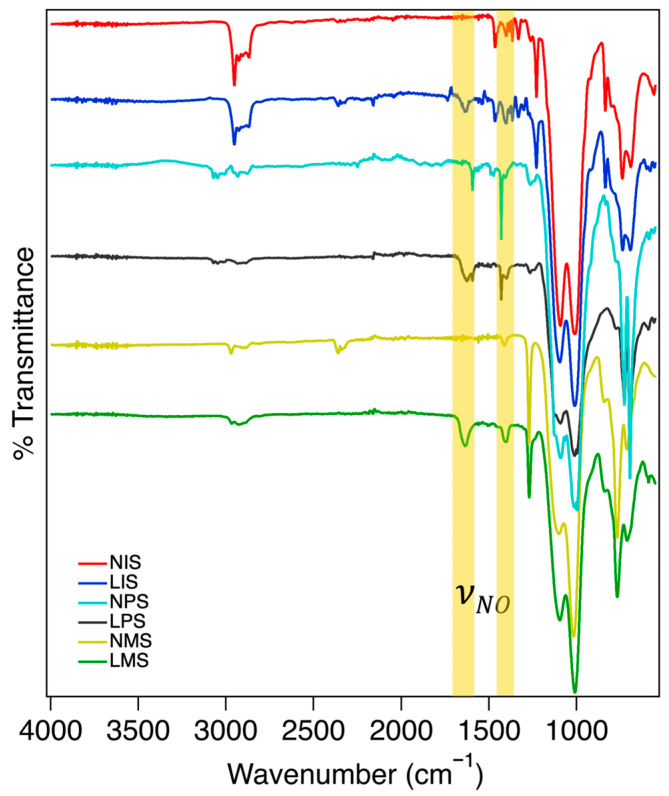
Comparative FTIR of loaded vs. non-loaded samples showing NO peak appearance in the range of 1550–1580 cm^−1^.

All resulting gels were characterized using FTIR to identify chemical markers and confirm NO loading (Figure 2). All preloaded organogels showed C-H peaks at ~2900 cm^−1^ and prominent silicon–oxygen double peaks between 1000 and 1100 cm^−1^. The BTSE bridge molecule is indicated by the small C-O peak ~1250 cm^−1^. Peaks around 1400 cm^−1^ indicate differences in the carbon–hydrogen stretching modes of the R groups in NMS, NIS, and NPS. Increased peak sizes and variation in the NIS and NPS at the 1400 cm^−1^ and 2900 cm^−1^ regions provide evidence for the presence of an isobutyl or phenyl substituent. Analysis of the NO-loaded samples showed the appearance of a peak at ~1600 cm^−1^ and the increase of 1480 cm^−1^ in the peak, consistent with the presence of an NO group bound to the thiol [34,35].

### 2.2. Solid-State MAS ^29^Si NMR of Organogel Networks

**Figure 3:**
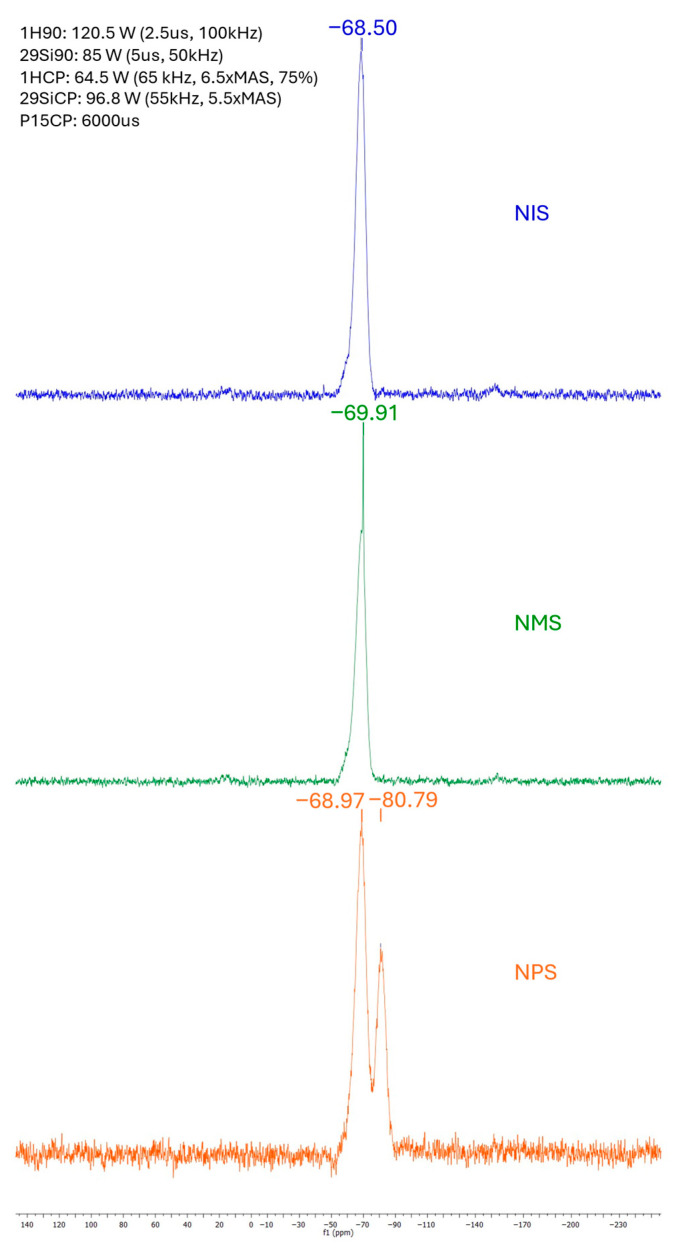
^29^SiNMR graphs showing the structure of SQs NIS, NMS, and NPS. All samples appear to be well-condensed T3 SQs, with very little T2 partially condensed shoulders present.

Solid-state 29Si NMR was used to evaluate the silicon structural composition and verify complete condensation of the alkoxysilane components (Figure 3). All samples showed peaks at 68–69 ppm, indicating a silsesquioxane polymeric system with three oxygen–silicon bonds and a single R group connected to a central silicon (T3). Small shoulders on the left of the central peak may indicate the presence of a small amount of incompletely condensed material where an ether or hydroxyl group is bonded to the central silicon atom instead of the Si-O-Si sequences (T2). On the NPS 29SiNMR plot, a secondary peak appears at 80.79 ppm, showing the presence of the phenyl R group adjacent to the central silicon [36,37]. Overall, these data showed the expected structures formed by the fluoride-catalysis process, and the absence of additional peaks indicates that the reaction proceeded to completion and did not produce off-target structures.

### 2.3. Characterization of the Thermal Behavior in Modified Elastomeric Samples

**Figure 4:**
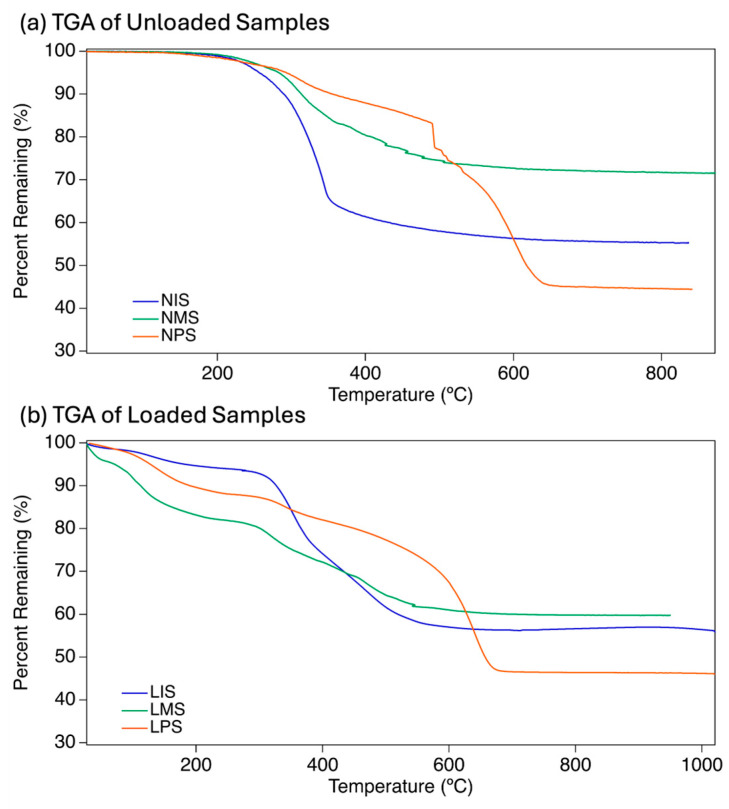
(**a**) TGA of unloaded samples with temperature gradient from 25 to 1000 °C. Initial sulfur loading is indicated by the downward slope from 250 to 300 °C, with substituent and bridge molecules burning off from 300 °C to the ceramic yield flattening of each sample. (**b**) TGA of samples post NO loading with temperature gradient from 25 to 1000 °C. NO loading is indicated by the slope from 25 to 100 °C, with subsequent volatilization of the remaining molecules until individual ceramic yields are observed at the areas of zero slope. This is consistent with the theoretical masses based on the mmol quantities utilized. Significant drops in percentage from 300 to 650 °C are indicative of R-group substituents and bridge sections being burned off, and a stuttering or backtracking slope indicates temperatures at which pores opened and cooler gases were released while the substituents were burned away [22]. A second set of TGAs (Figure 4b) was taken post-loading to analyze the amount of NO present on loaded samples. LIS showed approximately 4.3% loading with its slope from 25 to 123 °C. LPS released approximately 4% NO between 25 and 116 °C, indicating a high loading rate for the phenyl derivative. LMS NO release equated to 3.6% per the initial mass decrease between 25 and 45.5 °C, showing it to have the lowest loading of the three samples. This coincides with the low NO release rate and duration observed in the LMS samples shown later.

Thermal Gravimetric Analysis (TGA) is commonly used in polymer chemistry to find the composition and proportions of polymer chains and is especially useful for silicon-containing materials [38]. It shows the overall ceramic yield of the silica backbone remaining and allows us to determine the breakdown temperature of organic components and the release weight of volatile elements from the gel (i.e., NO) [33]. Sample ceramic yields were consistent with the expected masses of silica remaining after pyrolyzing the R-group molecules (Table 2). Unloaded samples showed a thiopropyl release from ~250 to 300 °C, equating to approximately 6% of the total mass for NMS and NIS and 4% total mass for NPS (Figure 4a).

### 2.4. Characterization of Gel Structure via SEM, EDX, SSA, and SAXS

**Figure 5:**
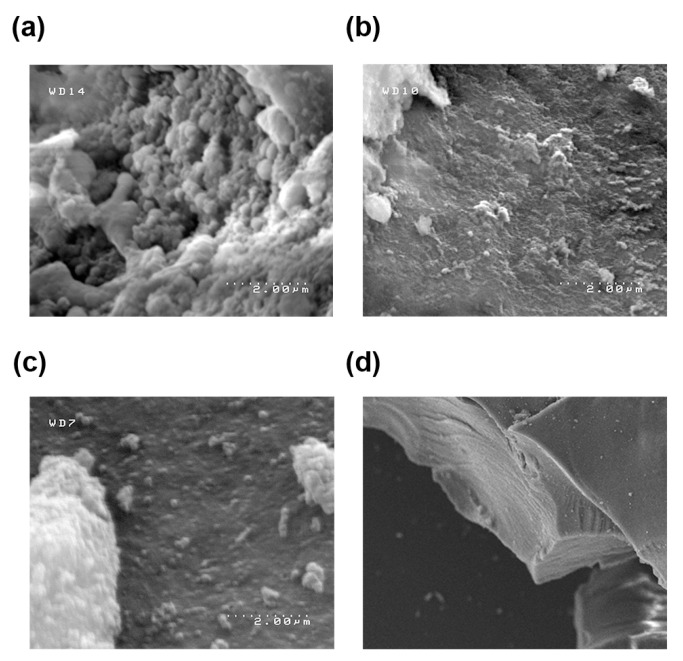
SEM image of (**a**) NMS at 2.00 μm under aperture 4, (**b**) NIS at 2.00 μm under aperture 4, and (**c**) NPS at 3.33 μm under aperture 4. (**d**) NPS striated side view under 200 μm and aperture 4.

To study the structural characteristics of each preloaded sample, scanning electron microscopy (SEM), energy-dispersive X-ray (EDX) analysis, and specific surface area (SSA) analysis were used to better visualize the polymer cages formed. All products were examined with SEM and a 2–200 μm resolution to visualize their surface structures. NMS appeared to have an amorphous, blobby surface, resulting in a high level of exposed surface (Figure 5a). This corresponds to the faster release of NO, as the releasing molecules can readily move through these amorphous structures and access more RSNO groups. NIS has a flatter, smoother surface than NMS, indicating that its large surface area is concealed within smaller pores that are shielded from NO-releasing agents (Figure 5b). NPS showed a smooth surface pockmarked with holes (Figure 5c). The NPS particles had flat layers stacked on top of each other to create striated edges, indicating a more uniform gel synthesis that prevented the formation of a surface area as large as NIS (Figure 5d). EDX results showed the expected silicon, oxygen, carbon, and sulfur peaks in each sample, and elemental analysis indicated a rough estimate of 4–6% sulfur integration in each sample. EDX molecular proportion estimations involving Si and C are low accuracy due to the presence of Si and C in the equipment, but multiple scans yielded consistent readings close to theoretical expectations.

Specific surface area (SSA) analysis was performed on each sample, and the resulting surface area and pore-size distributions were obtained (Table 3, Figures S1–S3). Consistent with the SEM images, the BET and DFT pore-size models showed NMS to have the largest average pore size (5.1 nm) but a relatively low SSA of ~160 m^2^/g, which we expected would lower the overall NO release. The average pore size on NIS is ~4 nm, consistent with a larger R group than methyl. This pore size, along with the higher hydrophobicity, was expected to further protect against initial buffer exposure bursts in loaded samples (LISs), and the higher SSA of NIS (559 m^2^/g) would result in the highest ability to load NO. Somewhat surprisingly, NPS BET analysis showed no porosity, as verified across multiple batches and tests, likely due to pore blocking by the phenyl ring size [33]. Due to this, we anticipated that this material would perform worse in NO loading and release versus the other two derivatives. However, this was not the case, as shown by the NO results below, which indicated that it had the best overall performance. This means that SSA and NO loading/release are likely decoupled, and surface/swelling may make a greater contribution to loading/release. Swelling data for NPS is shown in Figure S4, which shows an average wt% swelling of ~67%, suggesting that NPS shows high buffer interactions, whereas NMS and NIS offered little to no buffer swell (<5%). This further shows that LIS/LMS versus LPS systems may release NO under different mechanisms.

To further develop an understanding of the porosity in the systems and provide further structural evidence, small angle X-ray scattering (SAXS) was employed. SAXS spectra are shown in Figures S5–S8. Guinier analysis of the data shows non-linear behavior in the low-q range, suggesting that the samples contain aggregates [39]. This makes Rg determination less reliable in this region for all samples; however, the spacing data determined for NMS and NIS of 4.3 ± 0.2 nm and 4.8 ± 0.2 nm, both with 0.99 R2 values, respectively, are in the same range as that found using SSA DFT pore-size analysis. Guinier analysis of the NPS sample resulted in an Rg of 1.62 ± 0.08 nm but showed much lower confidence (R2 of 0.77). In the Guinier region, the NPS also showed lower scattering overall, suggesting that the particles themselves were less spherical, correlating with the SEM images. Conducting Bayesian Indirect Fourier Transform (BIFT) analysis [40] with BioXTAS Raw analysis software 2.4.1 [41] on these data sets allowed for the better overall determination of potential Rg values, radius distributions (P(r) vs. r), and Dmax values, which specify the maximum spacing between electrons found in a particle. Figures S6–S8 show this data and fitting, with Table 3 giving the Rg from (P(r)), Dmax, and χ2. For the NMS and NIS samples, the values are reminiscent of the Guinier-determined Rg values. The Rg value determined for NPS is 8.11 ± 0.01 nm, which is quite different from the Guinier-determined value. This mismatch is either reminiscent of the data itself being difficult to fit (note high χ2 of 2) or that the pore radii are much larger than that found for NMS and NIS. These radii would correspond to distances of ~16 nm, which should still fall within the mesoporous range, suggesting that pore accessibility may be more of an issue for the NPS sample and not that it is non-porous in nature. Regardless, the NPS sample shows the best performance overall for NO release, so even with a poor determined surface area, NO can still be removed effectively.

### 2.5. Mechanical Properties of Silicone Elastomers vs. Filled Composites

#### 2.5.1. Dynamic Mechanical Analysis

**Figure 6:**
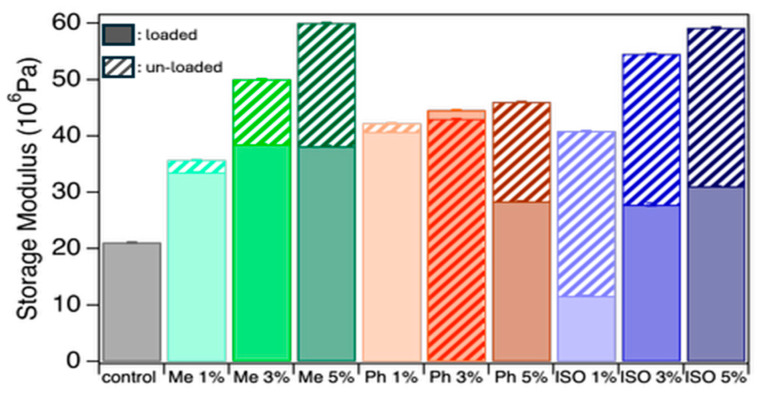
Variation in storage modulus of the control elastomer compared to Me, Ph, and ISO samples under loaded and unloaded conditions, highlighting the effects of gel incorporation on mechanical properties. The indicated loading levels (1, 3, and 5%) correspond to the weight percentage of gel incorporated into the elastomer.

The NO-loaded organogel materials were loaded into silicone resins at three different weight percentages (1, 3, and 5 wt%); trials at higher loadings (7 and 10%) negatively affected the silicone’s curability, which curtailed further investigation of these higher loadings. Loading the gels with nitric oxide (NO) decreased the storage modulus, indicating a slight softening of the samples upon NO incorporation (Figure 6) [42]. However, the addition of gels into the elastomer matrix increased the storage modulus across all compositions, suggesting improved stiffness due to gel reinforcement [43]. Unloaded NPS-composite samples showed a moderate increase in the modulus with increased gel loading, suggesting an almost plateau effect. At the same time, NIS-composite samples varied more between the 1, 3, and 5% loadings, particularly under unloaded conditions. An exception was observed with the LIS-composite 1% sample, which showed a reduced storage modulus. This deviation may be attributed to LIS’s tendency to self-aggregate, forming clumps within the elastomer matrix [44]. Such inhomogeneity likely contributes to the formation of visible pores, as seen in side-view images, which can compromise mechanical integrity. Overall, increasing the unloaded gel content (1%, 3%, and 5%) in the elastomer consistently increased the storage modulus, whereas NO-loaded composites were less consistent, possibly due to insufficient drying.

#### 2.5.2. Hydrophobicity/Hydrophilicity Evaluation

**Figure 7:**
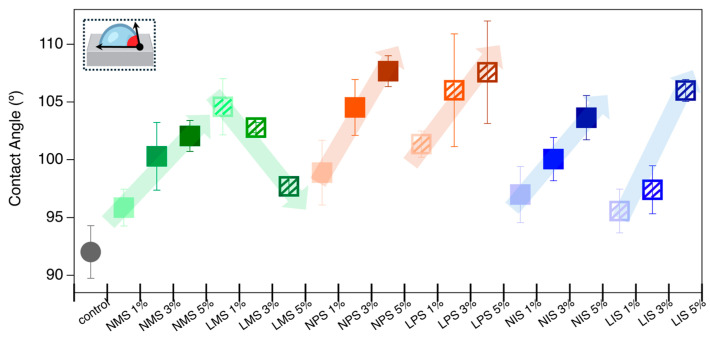
Contact angle measurements of water droplets on the surface of the control elastomer and elastomer samples incorporated with 1%, 3%, and 5% of unloaded (filled squares) and NO-loaded (hatched squares) Ph, Me, and ISO gels. The data illustrate the changes in surface wettability as a function of gel type, concentration, and NO loading. Green = methyl group, orange = phenyl group and blue = isobutyl group.

This study aimed to evaluate the effect of incorporating gel additives into an elastomer matrix and to assess the influence of nitric oxide (NO) loading on the wettability of the resulting composites [45]. Static contact angle measurements were used to determine the hydrophobic or hydrophilic nature of each sample upon exposure to a water droplet. A notable increase in contact angle was observed for all gel-containing samples compared with the control elastomer (Figure 7, Figures S9 and S10, indicating enhanced hydrophobicity [46]. In general, increasing the gel content within the elastomer led to a progressive rise in the contact angle, suggesting greater surface hydrophobicity [47]. However, an exception was observed with samples containing LMS gels, which exhibited an inverse trend: contact angles decreased with an increasing LMS concentration. This outlier could be the result of the non-loaded methyl group’s nonpolar contribution to the elastomer’s hydrophobicity, which was vastly overmatched by the NO’s polar contribution. This allowed the contact angle changes to be driven more by the amount of NO loaded into the system rather than by the R group’s nonpolar nature. Despite this, the contact angle of the 5% LMS-loaded sample remained higher than that of the untreated control, indicating a net increase in hydrophobicity.

#### 2.5.3. Nitric Oxide Release: Chemiluminescence Testing

**Figure 8:**
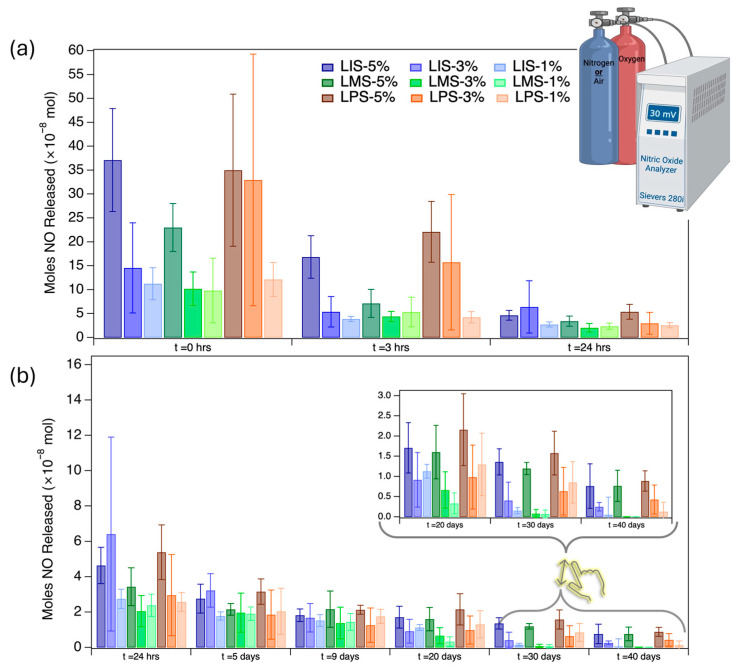
The changes in average NO release (×10^−8^ mol) were monitored at multiple timepoints, including (**a**) 0, 3, and 24 h, followed by (**b**) periodic measurements up to 40 days (N = 4). All samples were maintained at physiological conditions both during testing and during the incubation between testing timepoints.

A nitric oxide analyzer (NOA) was used to quantify NO released from circular, punched-out sample disks of the composite systems. Each disk had a diameter of 4 mm and possessed an area of ~0.82 cm^2^. Measurements were taken accurately at the specified intervals to determine NO release rates. The obtained data on NO from the samples are graphically plotted as the moles of NO released (×10^−8^ mol) as a function of time (Figure 8) for each sample type and percentage. Nitric oxide (NO) release in this work is reported in units of moles. Although alternative normalization units, including µmol/mg, are commonly used in the literature, direct comparisons are limited [48]. However, comparison with a recent study reporting NO release in units of µmol provided a useful reference point and indicates that the values obtained here are comparable with those reported in the literature [49]. Therefore, the data are presented in their original units. While the NO-release values fall toward the lower end of reported systems, the simplicity of the network design of the gels and the elastomer system and the sustained NO release over multiple days represent the key advantages of these materials, especially if loading can be increased.

For all samples, NO at 0 h was notably the highest, followed by a rapid decay after 3 h. This indicates that initial contact with the PBS buffer rinses the sample, releasing most of the surface NO on the exposed gel particles. The 5% LMS and 3% and 5% LPS samples show markedly elevated NO release at 0 hr but still showed sustained release over time. The NO release profile of all samples exhibited an initial burst, followed by a substantial decline in the release rate. After 24 h, release levels stabilized and remained relatively consistent within the error range for the next 20 days, with observable NO release still present after 40 days in higher-loading 3%+ samples, except LMS.

The pronounced initial release suggests that pre-soaking samples in phosphate-buffered saline (PBS) for 12–24 h may be beneficial before further testing; however, even the initial bursts would not be considered extremely high [50]. After the initial burst, all formulations maintained steady release rates, with LIS and LPS samples sustaining values within a range of 2–38 × 10^−8^ mol for at least 20 days across all weight loadings (Figure 8 and Figures S11–S15). The total amount of NO released in this study (2–38 × 10^−8^ mol) is consistent with values reported for NO-releasing polymer systems in the literature, supporting the potential physiological relevance of the observed release levels, albeit on the lower end [51]. The NO release profile is demonstrated by representative NOA traces collected at various time intervals (Figure S14). While LMS formulations depleted their NO payload more rapidly, the 5 wt% LMS sample continued releasing NO beyond 30 days, indicating potential suitability for applications requiring a shorter duration and reduced total NO output. Variability in measurements is expected to decrease with additional diethyl ether washing steps and the continued optimization of analytical protocols.

Analysis of mechanical properties revealed that nitric oxide (NO) loading significantly affected the material’s characteristics. In most cases, NO-loaded samples exhibited reduced stiffness, as evidenced by lower storage moduli than in unloaded samples. Furthermore, NO loading affected surface wettability, resulting in measurable changes in the water contact angle and indicating a slight shift in the materials’ hydrophobic/hydrophilic balance. No noticeable migration of the gel particles from the silicone resins was observed, suggesting material stability. The slightly lower hydrophilicity and possibly lower stiffness of the LPS system led to better sustained, higher NO release throughout the experiment. While this is not a surprising result, the fact that the NPS/LPS was non-porous yet still outperformed those with a higher loading capacity based on higher porosity was surprising, suggesting that porosity is much less important than the potential for the hydrophobic protection of NO-release sites.

## 3. Materials and Methods

### 3.1. Materials

Methyltriethoxysilane [MeSi(OEt)_3_] and iso-butyltriethoxysilane [(CH_3_)_2_CHCH_2_Si(OEt)_3_] were obtained from Gelest Inc., Morrisville, PA, USA. 3-Thiopropyltriethoxysilane [HSCH_2_CH_2_CH_2_Si(OEt)_3_], Tetra-n-butylammonium fluoride [(CH_3_CH_2_CH_2_CH_2_)_4_N^+^F^−^] (TBAF), t-butyl nitrite [(CH_3_)_3_CONO], deuterochloroform [CDCl_3_], sodium chloride, potassium chloride, potassium iodide, anhydrous ethyl ether, and glacial acetic acid were purchased from ThermoFisher Scientific, Waltham, MA, USA. 1,2-bis(triethoxysilyl)ethane [(EtO)_3_SiCH_2_CH_2_Si(OEt)_3_] (BTSE) was purchased from Accela ChemBio, San Diego, CA, USA. Acetonitrile, N,N-dimethylacetamide (DMAC), sodium phosphate dibasic, potassium phosphate monobasic, and sodium nitrite were from Sigma Aldrich, Saint Louis, MO, USA. N_2_ and argon were obtained from Linde, Danbury, CT, USA. Oomoo 30 Tin Cure Silicone Elastomer kit from Smooth-On Inc., Macungie, PA, USA, was used to contain/encapsulate the synthesized gels as composites.

### 3.2. Synthesis Methods

#### 3.2.1. Silsesquioxane Hybrid Organogel Synthesis

In a 500 mL round-bottom flask, 200 mL of ACN solvent was combined with 0.75 mL of deionized H_2_O. An aliquot of the appropriate mass of -RSH [thiopropyltriethoxysilane (TPTES)] and bridge molecule [BTSE, bistriethoxysilylethane], as given in Table 3, was then added and stirred for 5 min at room temperature. A total of 0.18 mL of TBAF was added to the solution, which was then stirred and stoppered for 12 h to undergo a fluoride-catalyzed sol–gel process. The white/off-white precipitate gel was collected by vacuum filtration and dried in a vacuum oven at 60 °C for 24 h. Once the initial product was dried, it was ground to a fine powder in a mortar and pestle and returned to the oven for at least two additional hours to remove any additional moisture. The procedure was also conducted by replacing methyltriethoxysilane (MTES) with iso-butyltriethoxysilane (IBTES) and phenyltriethoxysilane (PTES) at equivalent mmol amounts. All powder products were stored in the freezer in sealed containers to prevent moisture build-up and inadvertent degradation.

#### 3.2.2. NO Loading and Elastomer Impregnation

A total of 200 mg of each gel sample was placed into a 250 mL round-bottom flask with a stir bar. Each sample was treated with 10 mL of DMAC and 1 mL of t-butyl nitrite for 1 h or until the solvent had completely evaporated [7]. The product powder was filtered and washed with diethyl ether to ensure the complete removal of residual solvents. The products were transformed from bright red (MTES), orange (PTES), or pink (IBTES) back to their original white powder color after rinsing. To limit unintended NO release between analysis and impregnation, all products were stored at −20 °C, covered in foil, and in the dark.

Three weight proportion versions of the impregnated elastomer were created for each sample. Oomoo-30 tin-cured elastomer was used to develop SQ–silicone composites for the NO-release organogels. Note that tin-cure chemistry was used, as hydrosilylation with platinum was severely hindered by the presence of sulfur in the gel. Oomoo resin (Part A) and curing agent (Part B) were combined in a 1:1 mass ratio and transferred into pre-labeled vials (0.5 g each). To each vial, 1% (0.011 g), 3% (0.033 g), or 5% (0.055 g) of the loaded NO-release gels was added, and the mixture was thoroughly mixed to ensure homogeneous dispersion within the polymer matrix. Following mixing, the vials were left to cure for 72 h under ambient conditions in a dark fume hood. After complete curing, a 4 mm circular punch was used to extract cylindrical samples of uniform dimensions (4 mm × 4.5 mm). The resulting sample disks were sealed and stored at −20 °C until further analysis.

### 3.3. Characterization Methods

#### 3.3.1. Fourier Transform Infrared Spectroscopy Analysis (FTIR)

All products were analyzed using a Thermo Scientific Nicolet iS5 transform infrared spectrometer (Waltham, MA, USA). The samples were placed on a ZnSe crystal and scanned from 500 to 4000 cm^−1^ in 32 scans with a data spacing of 0.121 cm^−1^.

#### 3.3.2. Thermal Gravimetric Analysis (TGA)

Pre- and post-NO-loading samples were tested for ceramic yield and thermal stability using a Hitachi STA7200 Thermal Analysis System (SN:19112035C1-01) and NEXTA Software 2.0 (Hitachi High-Tech Science Corporation, Tokyo, Japan, 2018, 2019). Sample masses (mg) used in the experiments are listed in Table 2. Samples were analyzed in an alumina pan from 25 to 1000 °C at a heating rate of 10 °C min^−1^ with 60 mL min^−1^ of air flow.

#### 3.3.3. Specific Surface Area (SSA) and Porosity Analysis

All non-loaded powders were analyzed by a Micrometrics 3FLEX surface and catalyst characterization analyzer (Micromeritics Inc., Norcross, GA, USA). Approximately 350 mg of the ground and dried sample was analyzed at −196 °C (73 K) under N_2_ to obtain adsorption/desorption isotherms. The mesopore data were determined using a multipoint method with 12 data points at 0.050 (p/p0) relative-pressure increments, starting at a relative pressure of 0.8 (p/p0). Brunauer–Emmett–Teller (BET) and DFT pore methods were used to assess surface area and pore size.

#### 3.3.4. Solid State MAS ^29^Si NMR

All samples were analyzed using a Bruker 600 MHz Avance III HD DNP spectrometer (Billerica, MA, USA) equipped with a 3.2 mm HXY-DNP probe. Samples were contained in 3.2 mm zirconium rotors and spun at 10 kHz (Magic Angle Spinning). All samples were kept at approximately 300 K during testing. ^29^Si cross-polarization (H-Si) experiments were performed using the same data set: 256 scans; 7 s recycle delay; and 30 min experiments. Tetramethylsilane (TMS) was used to calibrate pulse and chemical shift values.

#### 3.3.5. Scanning Electron Microscopy Analysis (SEM) with Energy-Dispersive X-Ray Analysis (EDX)

All samples were carbon-tape-loaded onto stubs and coated with gold–palladium wire using a Denton TT-IV high-vacuum coater. The stubs were then analyzed on a Hitachi S2700 scanning electron microscope (Hitachi High-Tech America, Schaumburg, IL, USA) from 15 to 20 kV using apertures 3 and 4 at working distances from 10 to 20. The instrument’s EDAx (EDAx Genesis detecting unit, model #PV77-47700-ME) attachment was used to determine the elemental makeup of each sample, estimate their sulfur proportions, and verify the presence of sulfur.

#### 3.3.6. Small Angle X-Ray Scattering (SAXS)

SAXS was conducted on powderized samples of NMS, NIS, and NPS using a Rigaku Ultima III high resolution XRD instrument (Rigaku Americas, The Woodlands, TX 77381, USA) in transmission SAXS configuration (University of Toledo Center for Materials and Sensor Characterization). A Cu Kα source (40 kV, 44 mA), a wavelength of 1.54059 Å, scanned over 0.109–7.989 degrees 2θ at a rate of 0.02°/100 s, and I(p) = 33,352/65 were used for analysis. Samples were placed in a plastic pouch for containment and inserted into a 1 mm stainless steel holder in transmission mode for analysis over a period of 12 h. Scans were averaged and the plastic pouch subtracted from the data. All data analysis was conducted with the BioXTAS RAW 2.4.1 software package using Guinier and Bayesian Indirect Fourier Transform (BIFT) analysis [41].

#### 3.3.7. Nitric Oxide Analysis (NOA)—Calibration

Nitric oxide (NO) release measurements were conducted using a gold standard Sievers 280i chemiluminescence Nitric Oxide Analyzer (GE Analytical Instruments, Boulder, CO, USA). During analysis, nitrogen was used as a carrier gas to interface with the NOA, which operated at a supply pressure of 6.2 Torr and a cell pressure of 5.7 ± 0.2 Torr. Data acquisition and visualization were performed using the LabChart (v8.0) software. Quantification of NO release was achieved by integrating the photomultiplier tube (PMT) emission signal from the NOA, which was calibrated with sodium nitrite (NaNO_2_) standards [52]. The amount of NO released was calculated using Equation (1).

(1) $$Moles\;of\;NO\;released=Area\;of\;peak\times (9.17\;\pm \;3\times 10^{-12})$$

**Figure 9:**
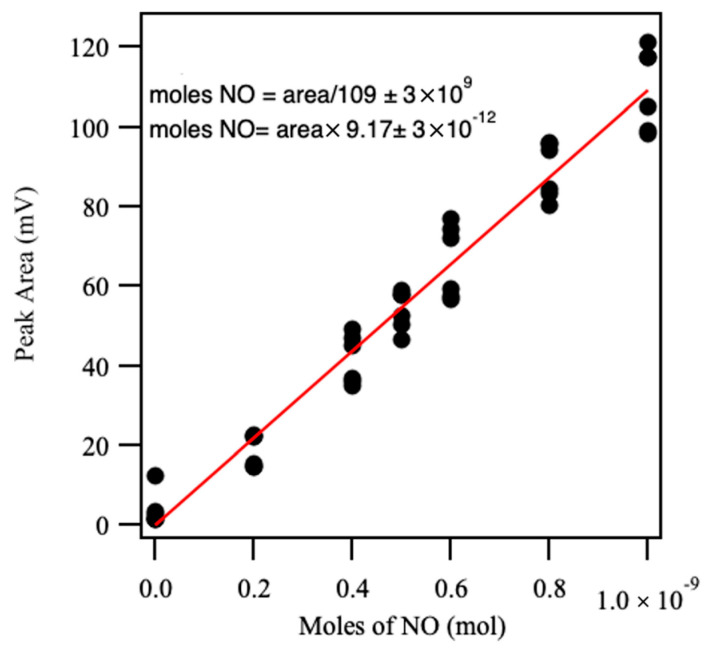
Calibration curve for the Sievers 280i NOA using sodium nitrite standards (20–100 µM). Peak area correlated linearly with NO concentration for accurate quantification.

Calibration was performed by injecting 10 µL aliquots of sodium nitrite solutions at concentrations of 20, 40, 50, 60, 80, and 100 µM into a purge vessel [53]. The vessel contained a reducing solution prepared by dissolving 75 mg of a reducing agent in 2 mL of water, followed by the addition of 6 mL of glacial acetic acid. This solution chemically reduced nitrite to nitric oxide (NO), which was swept into the NOA by the carrier gas. Inside the NOA, NO reacts with ozone to form electronically excited nitrogen dioxide (NO2), which emits light upon relaxation. The emitted chemiluminescence, proportional to the amount of NO present, was detected by the PMT and integrated over time [54]. The resulting calibration curve (Figure 9) allowed for accurate determination of NO release from test materials, where the slope of the calibration curve, with a value of (9.17 ± 3 × 10^−12^), was used as the conversion factor to relate peak area to the moles of NO released.

#### 3.3.8. Nitric Oxide Analysis (NOA)—Sample Analysis

Following the nitrosation of the -RSNO molecule, measurements of NO release commenced. Various elastomer samples were prepared with 1%, 3%, or 5% by weight of the -RSNO SQs. A 4 mm-diameter dot from each sample was promptly immersed in a flask containing 3 mL of PBS buffer at pH 7.4, and an immediate NO measurement was taken. The measurements were conducted at multiple timepoints, 0, 3, and 24 h, and 5, 9, 20, 30, and 40 days, to monitor the sample’s release profile at 37 °C. Throughout the study, the sample was stored in the dark in phosphate-buffered saline (PBS) at pH 7.4 and maintained at 37 °C to ensure uniform experimental conditions. If the sample continued to release a significant amount of nitric oxide, measurements and storage were extended to the subsequent interval. The measurements were integrated, and the moles of released NO were calculated using Equation (1).

#### 3.3.9. Dynamic Mechanical Analysis (DMA)

Mechanical properties of silicone elastomer composite samples were measured using a DMA8000 (PerkinElmer Inc., Hopkinton, MA, USA). Rectangular specimens with approximate dimensions of 29 ± 0.5 mm length, 12 ± 0.1 mm width, and a thickness of around 2 ± 0.1 mm were prepared and clamped in single cantilever geometry. Measurements were performed at 37 °C in phosphate-buffered saline (PBS, pH 7.4) to simulate physiological conditions. Experiments were conducted under a static force of 1.5 N and a strain amplitude of 0.1 mm. After an initial 10 min equilibration period, cyclic loading was applied over three cycles, each lasting 15 min. The storage modulus (elastic response) was continuously monitored to assess the effect of mechanical loading on the viscoelastic properties of silicone elastomer composites (NO-loaded and unloaded) under simulated physiological conditions.

#### 3.3.10. Water Contact Angle Measurement

Contact angle measurements were performed to evaluate the surface wettability of elastomer samples, including a control blank elastomer as well as NO-loaded and unloaded 1, 3, and 5 wt% compositions. A Zeiss Stemi 2000-C light microscope (Carl Zeiss Meditec Inc., Dublin, CA, USA) equipped with an AxioCam ERc5s camera (Carl Zeiss Meditec Inc., Dublin, CA, USA) was used at 0.65× magnification to capture images. Approximately one droplet of water (~10 µL) was deposited onto each sample surface using a syringe. For each composition, three samples were analyzed, and for each sample, the contact angle was measured three times by averaging the left- and right-side angles of the droplet, yielding a total of six measurements per composition. Images were analyzed using ImageJ software (version 1.54g) with the contact angle plug-in (manual points method), developed by the National Institutes of Health (Bethesda, MD, USA).

## 4. Conclusions

The use of variably substituted organogel silsesquioxanes as -RSNOs offers options to extend the duration of NO release and control release rates, as demonstrated with methyl-, phenyl-, and iso-butyl-functionalized systems. Changing the R-group substituent in these organogels alters polymer formation and inherent physical properties, thereby affecting buffer permeability into the polymer structure and interactions with RSNO groups. At present, LIS and LPS systems appear to offer the most promising long-term release characteristics, sustaining NO delivery well beyond a target of 30 days, with some samples continuing release for several weeks after. Collectively, the studied formulations demonstrate functional NO release as -RSNO systems, underscoring their potential as versatile delivery systems. Continued structural variation, substituent optimization, and systematic testing will be critical for refining their release rates and expanding their utility across diverse biomedical applications and may include the use of better-protected tertiary thiols rather than primary thiols. All samples could be subjected to solvent rinsing to reduce the large initial NO burst observed in higher-percentage LMS/LIS and all LPS samples, but altering the R-group and TPTES proportions could also help mitigate this burst and increase the release durations. LPSs’ initial burst is 20 times higher than expected (likely due to higher surface loading from lower porosity), but the layered conformation of the polymer extends the NO release duration at higher molar values. This shows decoupling of the specific surface area from release expectations. LMSs’ low initial burst and week-long duration could be helpful in specific applications. By leveraging simple sol–gel synthesis and the ability to alter molecules’ hydrophobicity, scientists can continue to develop more finely tuned SQs for a broader range of applications, and higher RSNO loadings may improve overall NO release. The extended duration will lead to longer-lasting products and, therefore, lower overall costs for medical institutions and patients.

## 5. Patents

USPTO Patent Application (63/808,061).

## Figures and Tables

**Table 1:** Monomer used to create SQ gels and naming legend of sample abbreviations. (R-group: green = methyl group, orange = phenyl group and Blue = isobutyl group).

R-Group	RSNO Mass (g)	R-Group Mass (g)	Bridge Mass (g)	N = Non-Loaded L = Loaded	Abbreviation
MTES	0.245	0.890	0.361	N	NMS
				L	LMS
PTES	0.245	1.199	0.361	N	NPS
				L	LPS
IBTES	0.245	1.099	0.361	N	NIS
				L	LIS

**Table 2:** Sample weights, T_5%_ temperatures, and ceramic yields when tested via TGA. (R-group: green = methyl group, orange = phenyl group and Blue = isobutyl group).

R Group	TGA Loading (mg)	Temperature at 5% Mass Loss (T_5%_)	Residue at 1000 °C (Ceramic Yield)
NMS	8.961	298 °C	71.6%
LMS	7.727	72 °C	59.8%
NPS	8.378	299 °C	44.4%
LPS	7.667	126 °C	46.4%
NIS	8.172	256 °C	55.3%
LIS	5.276	175 °C	56.7%

**Table 3:** Surface area and DFT slit pore analysis and SAXS-BIFT analysis of samples gives insight into the shape and negative space within the porous SQ structures.

R Group	Surface Area (m^2^/g)	Ave. Pore Size (Å)	R_g_ (nm)	D_max_ (nm)	χ^2^
NMS	160	51	4.3 ± 0.5	14.3 ± 0.6	1.29
NPS	0.5	N/A	8.11 ± 0.01	21.58 ± 0.05	2.05
NIS	559	40	4.4 ± 0.2	14.4 ± 0.5	1.68

## Data Availability

Data is available upon request from the corresponding authors or as electronic Supporting Information for this manuscript.

## Supplementary Materials

The following supporting information can be downloaded at https://www.mdpi.com/article/10.3390/molecules31081343/s1, Figures S1–S3: nitrogen adsorption/desorption-specific surface area analysis and DFT pore size distributions, Figures S4–S8: additional nitric oxide release data.
