# Supplementary material for: Network Silsesquioxane-Based Organogel/Silicone Composites for the Long-Lasting Delivery of Nitric Oxide
Source: Molecules. 2026 Apr 19;31(8):1343. doi: 10.3390/molecules31081343 (PMC13119455; doi:10.3390/molecules31081343)
Supplement: Supplementary file 1 [file molecules-31-01343-s001.zip › molecules-4198210-supplementary.pdf]

Electronic Supporting Information

Network Silsesquioxane-Based Organogel/Silicone Composites for the Long-Lasting Delivery of Nitric Oxide

Kyle D. Hallowell [1,†], Fatima Naser Aldine [1,†], Hope N. Vonder Brink [1], Ashley K. Mockensturm [1], Hitesh Handa [2,3], Elizabeth J. Brisbois [2], Alexis D. Ostrowski [1,*] and Joseph C. Furgal [1,*]

[1] Department of Chemistry and Center for Photochemical Sciences, Bowling Green State University, Bowling Green, OH 43403, USA

[2] School of Chemical, Materials, and Biomedical Engineering, University of Georgia, Athens, GA 30602, USA

[3] Pharmaceutical and Biomedical Sciences Department, College of Pharmacy, University of Georgia, Athens, GA 30602, USA

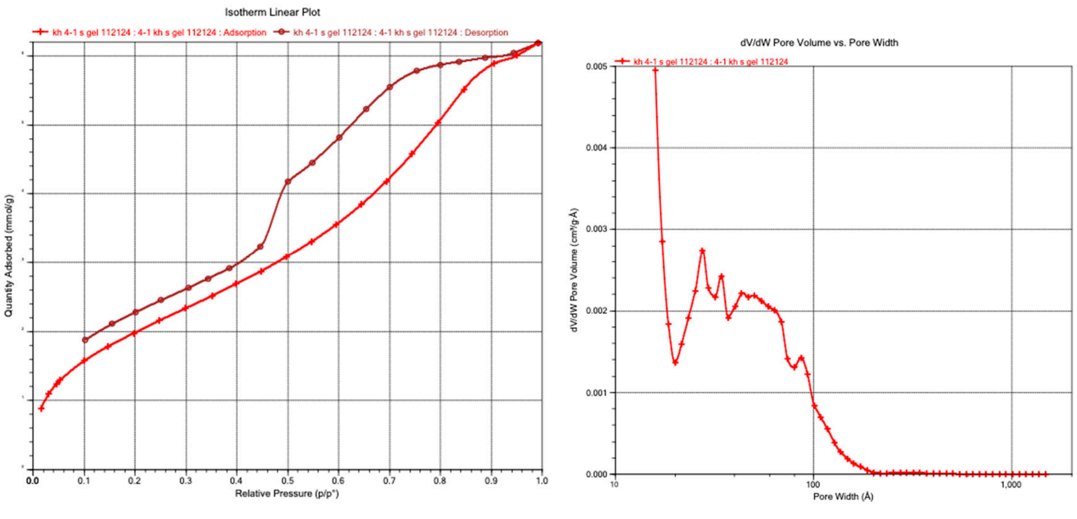

Figure S1. NMS Gel adsorption and desorption isotherms from BET and DFT pore size analysis.

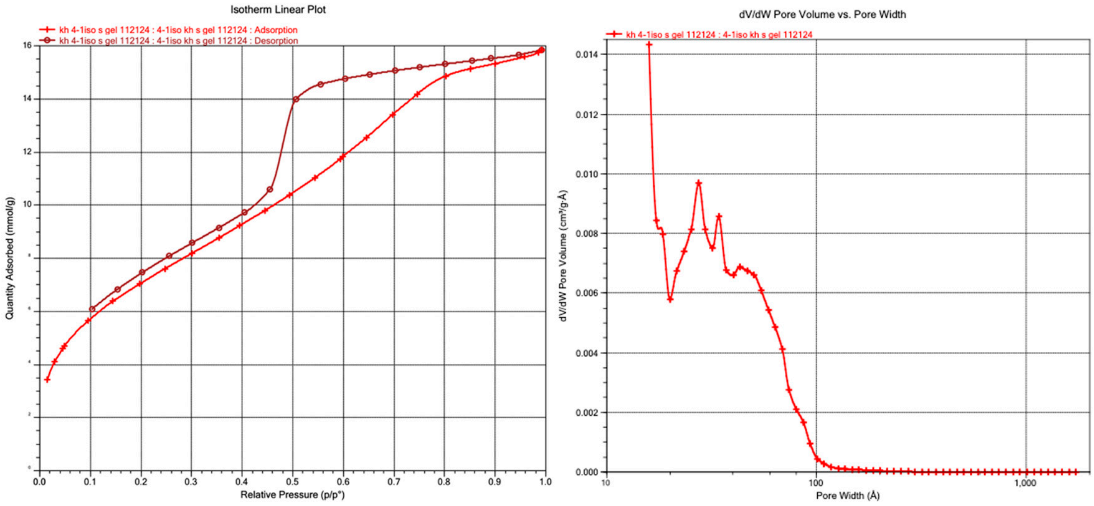

Figure S2. NIS Gel adsorption and desorption isotherms from BET and DFT pore size analysis.

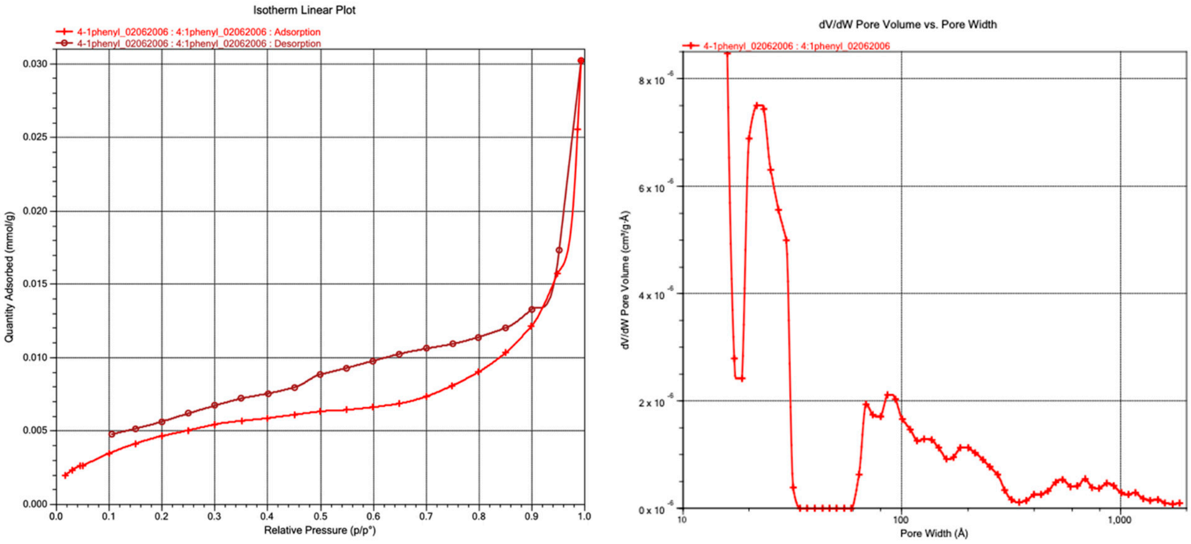

Figure S3. NPS Gel adsorption and desorption isotherms from BET and DFT pore size analysis.

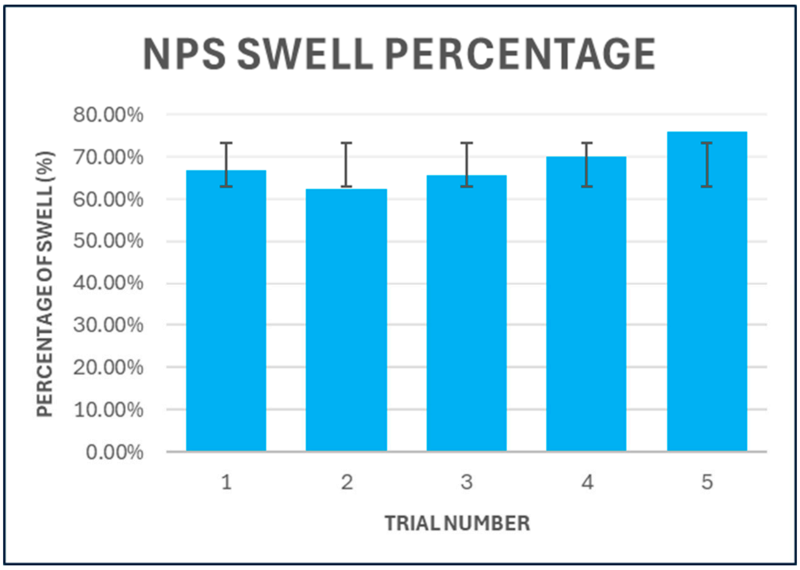

Figure S4: Swelling percent of ~0.05g of NPS when dosed with PBS buffer.

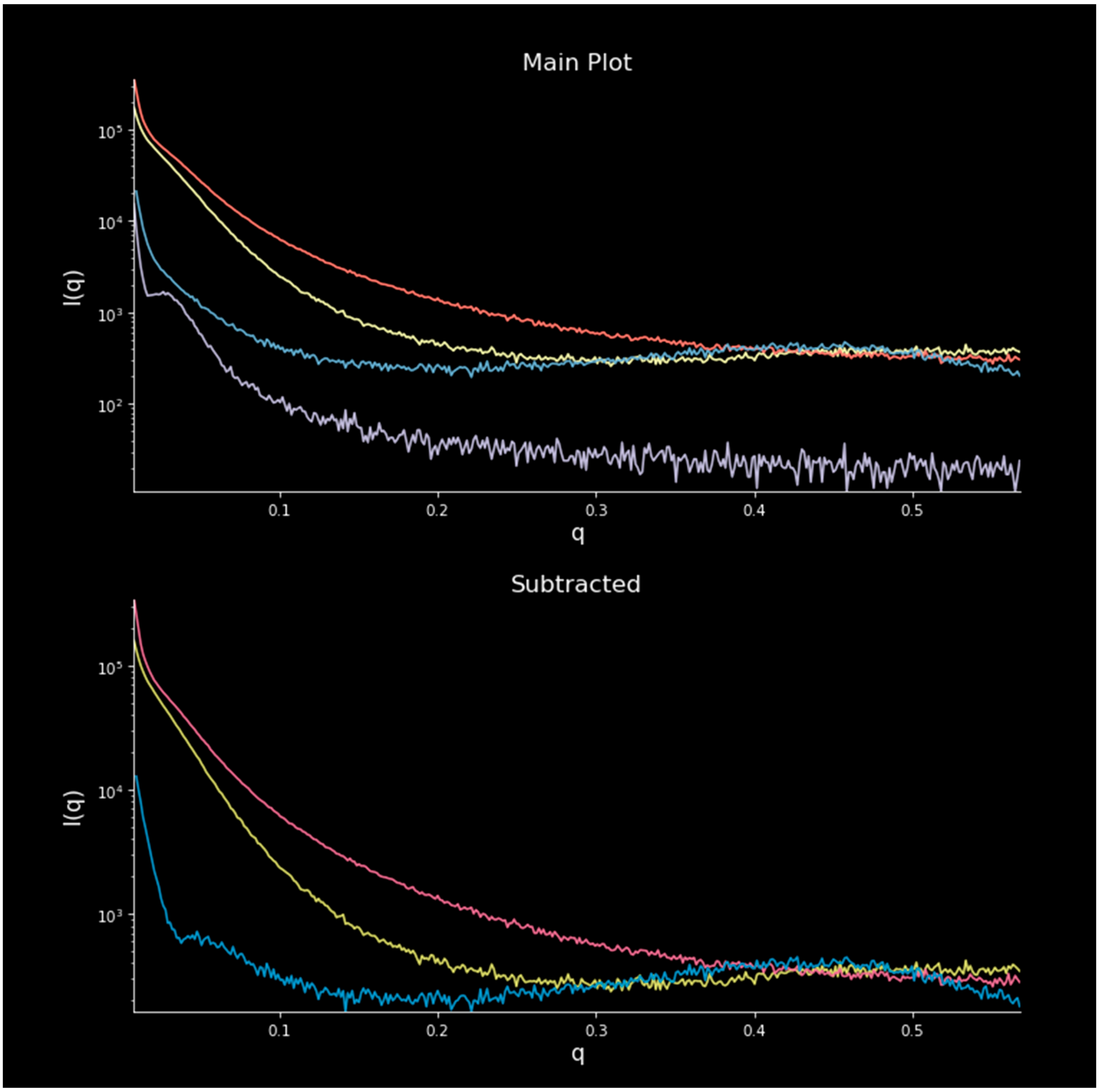

Figure S5. Small angle x-ray scattering (SAXS) data from NMS (pink), NIS (yellow), and NPS (blue) in intensity (I(q)) versus q (**Å**$^{-1}$). The plastic pocket used to contain the samples is given in purple and was subtracted from each of the samples. Plots and analysis conducted in BioXTASRAW software 2.4.1.

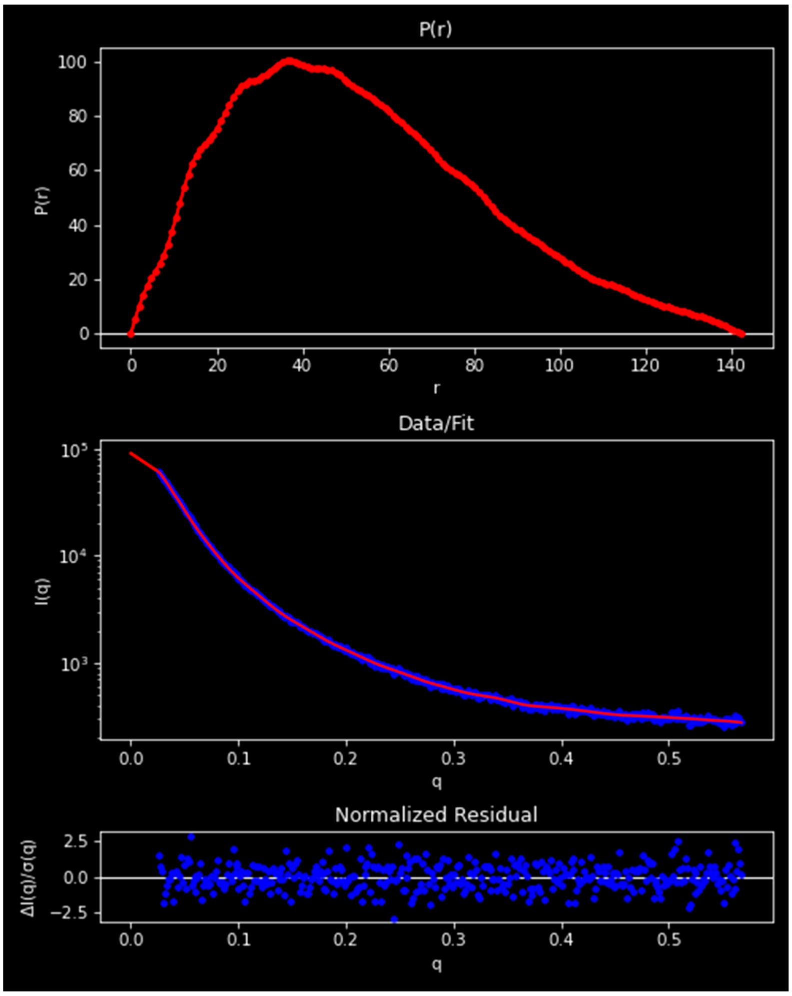

Figure S6. SAXS – BIFT (Bayesian Indirect Fourier Transform) method for the calculation of D_{max} (the longest dimension between particles, 14.3±0.6 nm) and R_g (root mean square distance from center of masses) for NMS sample set. R_g calculated from this method P(r) is in reasonable agreement with Guinier analysis of 4.3±0.5 nm versus 4.3±0.2. Note that aggregation causes non-linearity in the low q region and makes the BIFT analysis which focuses on high q analysis more reliable than Guinier analysis for these samples.

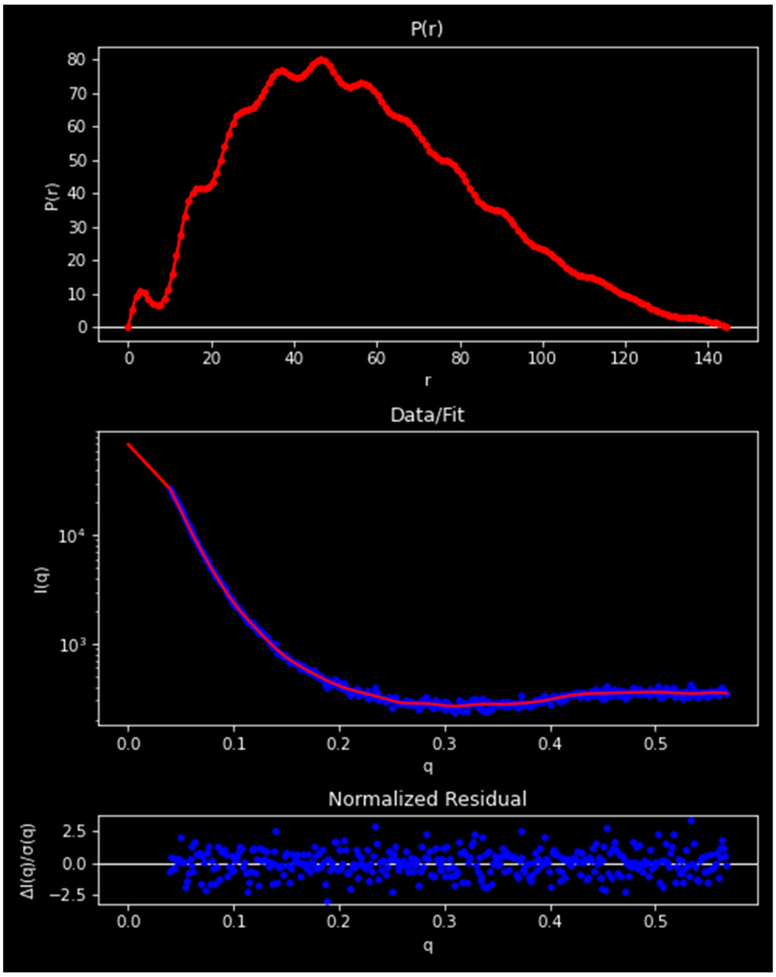

Figure S7. SAXS – BIFT (Bayesian Indirect Fourier Transform) method for the calculation of D_{max} (the longest dimension between particles, 14.4±0.5 nm) and R_g (root mean square distance from center of masses) for NIS sample set. R_g calculated from this method P(r) is in reasonable agreement with Guinier analysis of 4.4±0.8 nm versus 4.8±0.2 nm.

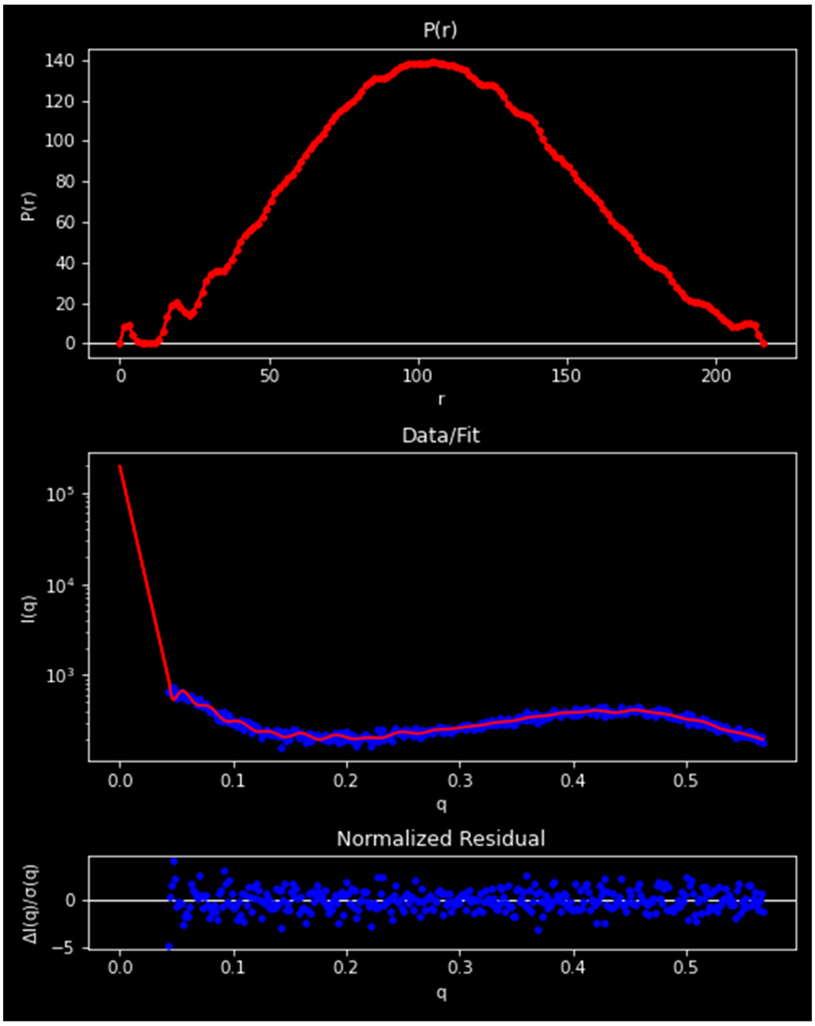

Figure S8. SAXS – BIFT (Bayesian Indirect Fourier Transform) method for the calculation of D_{max} (the longest dimension between particles, 21.58±0.05 nm) and R_g (root mean square distance from center of masses) for NPS sample set. R_g calculated from this method P(r) is in poor agreement with Guinier analysis, 8.11±0.01 nm versus 1.62±0.08 nm.

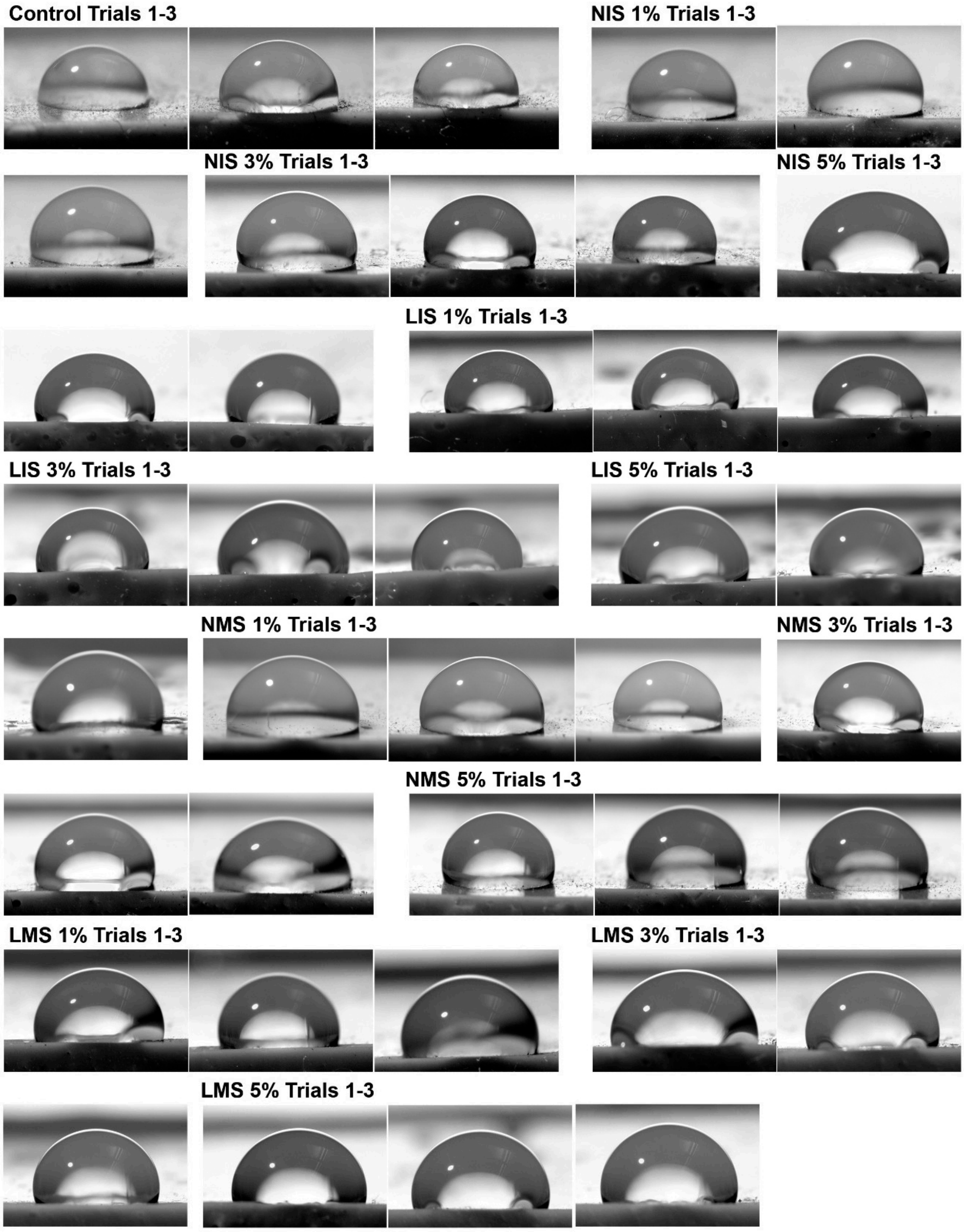
Control Trials 1-3
NIS 1% Trials 1-3
NIS 3% Trials 1-3
NIS 5% Trials 1-3
LIS 1% Trials 1-3
LIS 3% Trials 1-3
LIS 5% Trials 1-3
NMS 1% Trials 1-3
NMS 3% Trials 1-3
NMS 5% Trials 1-3
LMS 1% Trials 1-3
LMS 3% Trials 1-3
LMS 5% Trials 1-3

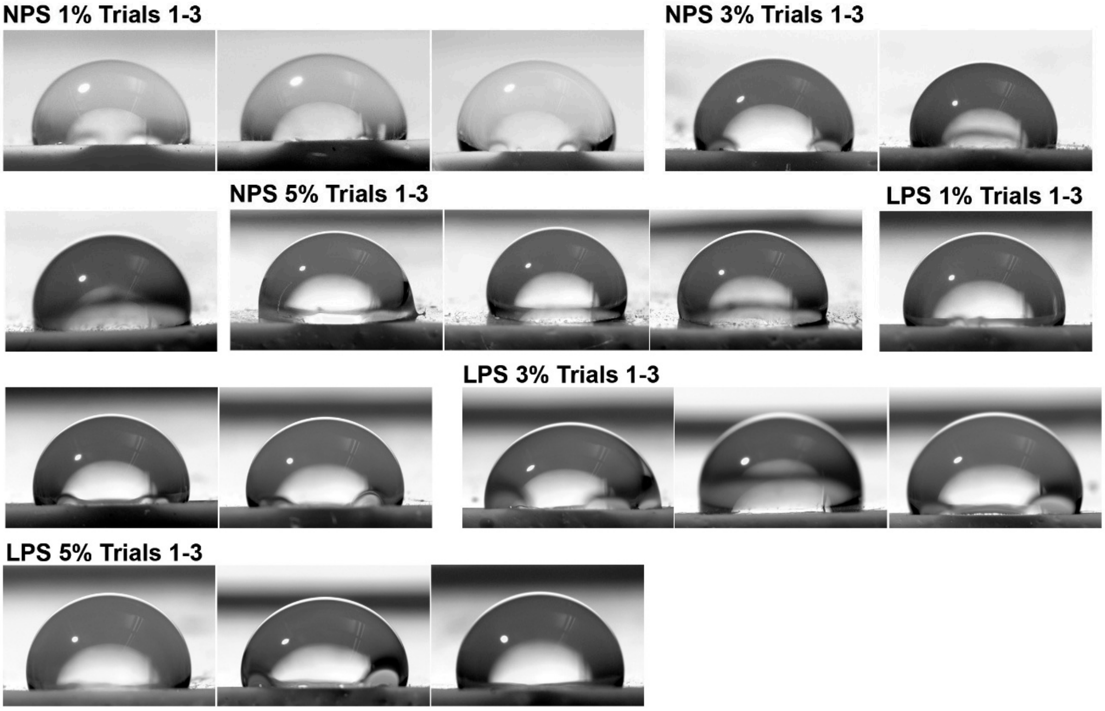

Figure S9-S10: Contact angle images used to find internal contact angles of three trials per sample type.

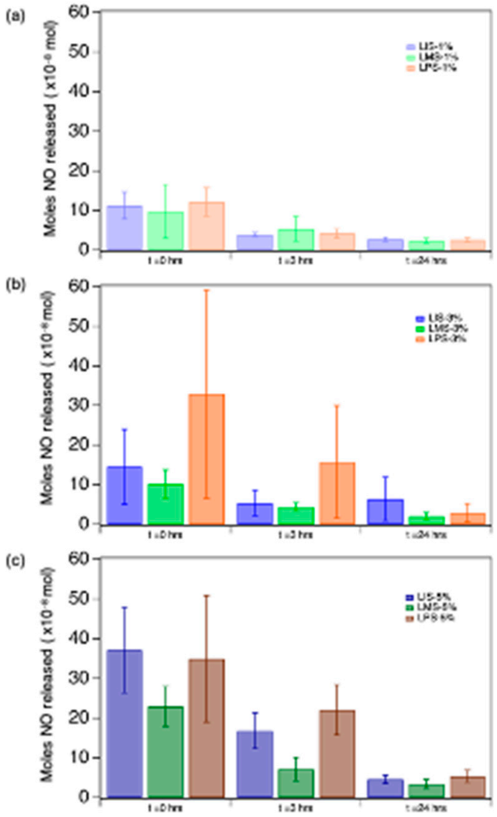

Figure S11: a) 1, b) 3, and c) 5% sample's released moles from 0-24 hours (N=4).

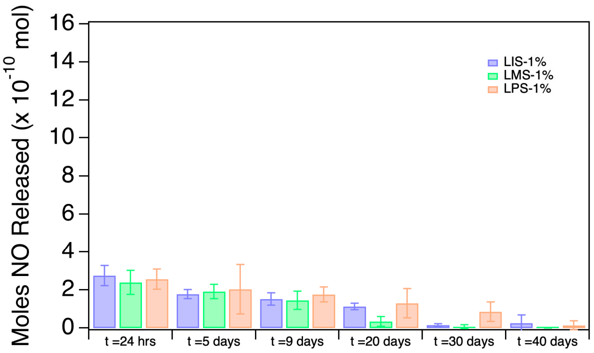

Figure S12: The 1% loaded sample release rates over 30 days. LMS and LIS samples released low amounts of NO and were considered depleted before the 30-day mark. LPS stayed consistent between 0.5-3 $\times 10^{-8}$ mol and continued to be monitored beyond 30 days (N=4).

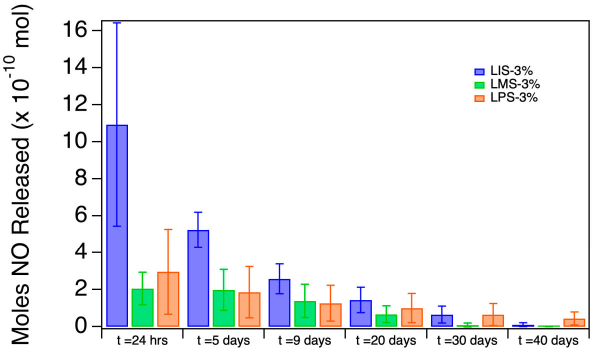

Figure S13: 3% loaded sample release rates over 30 days. LMS samples released low NO levels and were considered depleted at 20 days. LIS and LPS samples maintained notable NO release for the 30-day trial and beyond (N=4).

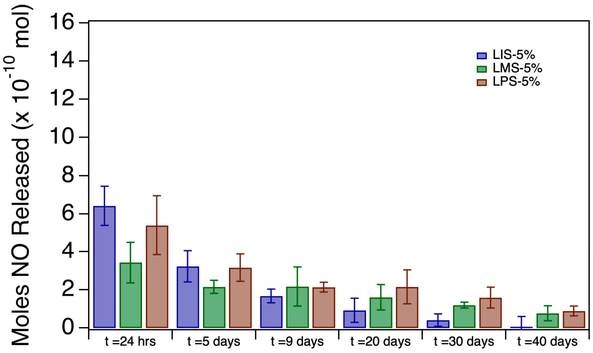

Figure S14: The NO of 5% loaded sample release rates over 30 days. All samples maintained acceptable NO release for the 30-day trial and beyond (N=4).

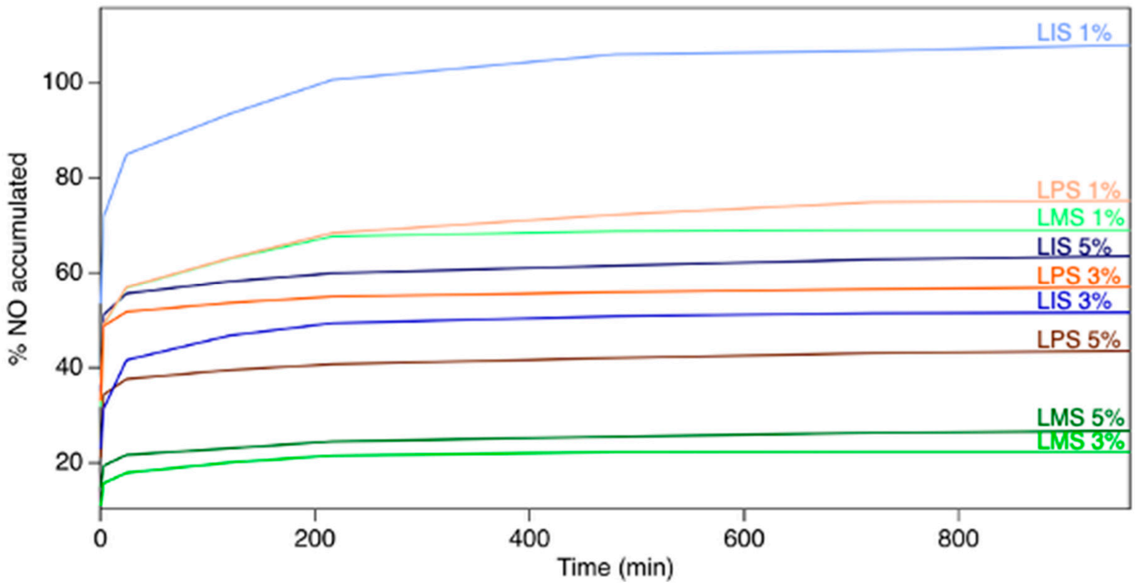

Figure S15: Percent of total calculated mols of NO remaining after 800 minutes of NOA treatments. This allows to calculate the total moles of NO released during testing.
